# An In-Vitro Analysis of the Surface Treatment of Orthodontic Bracket Bases With Er,Cr:YSGG Laser and Its Effect on Shear Bond Strength

**DOI:** 10.7759/cureus.44404

**Published:** 2023-08-30

**Authors:** Rebekah Raju, Ashwin George, Prasanna Aravind T. R.

**Affiliations:** 1 Department of Orthodontics and Dentofacial Orthopaedics, Saveetha Dental College and Hospital, Chennai, IND; 2 Department of Orthodontics and Dentofacial Orthopaedics, Saveetha Dental College and Hospital, chennai, IND

**Keywords:** adhesive interface, bonding, bond strength, mesh, orthodontic brackets, laser

## Abstract

Introduction

Shear bond strength is indispensable to prevent the debonding of orthodontic brackets. Lasers have been proven to alter the bond strength of orthodontic brackets, but their efficiency has not been validated in many trials. The study aimed to evaluate the impact of the Er,Cr:YSGG laser on the bases of orthodontic brackets and determine their bond strength with the enamel surface.

Materials and methods

The Waterlase iPlus (made in the USA in 2012), comprising an Er,Cr:YSGG laser, was used. Based on the surface treatment of brackets, two groups were assigned (n=10), comprising laser-treated and untreated bracket bases. The brackets were treated with the minimum laser intensity (50 Hz, 4.5 W). Then, the brackets of both groups were attached to the labial surfaces of previously extracted premolars, respectively. The shear bond strength of brackets (SBS) was assessed using the universal testing device, and the Adhesive Remnant Index (ARI) was also measured. An independent sample t-test was used to compare the bond strength between the laser-treated and untreated brackets.

Results

The mean bond strength of laser-treated and control group brackets was 5 MPa and 8.63 MPa, respectively. The laser-treated brackets showed lower bond strength than the control brackets, but the results were statistically insignificant (p=0.23). The ARI analysis stated that bond failures occurred mostly in the region of the bracket and adhesive interface.

Conclusion

Laser-etched bracket bases showed lesser shear bond strength than the untreated ones, though the difference was statistically insignificant.

## Introduction

Achieving the optimum bond strength for brackets is one of the major criteria for successful orthodontic treatment. Various methods such as milling, welding, brazing, chemical etching, or sintering have been used to enhance the retention of the adhesive to the metal bracket bases. In spite of these advancements, bond failure in brackets is one of the most common problems faced by clinicians. Recently, laser-structured bracket bases and metal plasma-coated bases have been used to further improve retention [1].

The use of innovative laser treatment enhances the specific area, improves wettability, and increases surface energy. Lasers are widely used in the field of dentistry for hard and soft tissue applications. Increased efficiency, specificity, simplicity, low cost, and comfort of dental treatment were all improved with the use of laser technology [2].

The most common lasers used in dentistry are erbium lasers, such as erbium-doped yttrium aluminium garnet (Er:YAG) and erbium, chromium: yttrium-scandium-gallium-garnet (Er,Cr:YSGG), as well as argon, carbon dioxide, diode, and neodymium-doped yttrium aluminium garnet [3]. With the progressive development of laser technology, erbium lasers have become increasingly important. Erbium lasers are currently used for a variety of dental applications [4]. Orthodontics has also utilised YSGG lasers to etch enamel, remove orthodontic adhesive, and debond the brackets [5].

One such minimally invasive dental laser device is the Waterlase iPlus (made in the USA in 2012), consisting of the Er,Cr;YSGG laser. It has been widely used in implants, hard tissue, soft tissue, and other crucial applications [6]. This laser system has not been used previously to determine the alteration of the bracket bond strength. Thus, the purpose of this study was to assess the impact of the Er,Cr:YSGG laser on orthodontic bracket bases and its effect on the shear bond strength.

## Materials and methods

Preparation of bracket bases

This in vitro study contained metal brackets (AO mini master standard edgewise brackets), which were segregated into two groups based on the bracket base’s surface treatment (n=10). The first group consisted of untreated brackets (the control group), and the second group consisted of laser-treated bracket bases. Based on the serious clinical implications of the laser on the metal surface, necessary precautions were taken during the procedure. Laser safety glasses were worn in order to prevent exposure to direct, reflected, or scattered laser radiation.

Laser treatment

**Figure 1:**
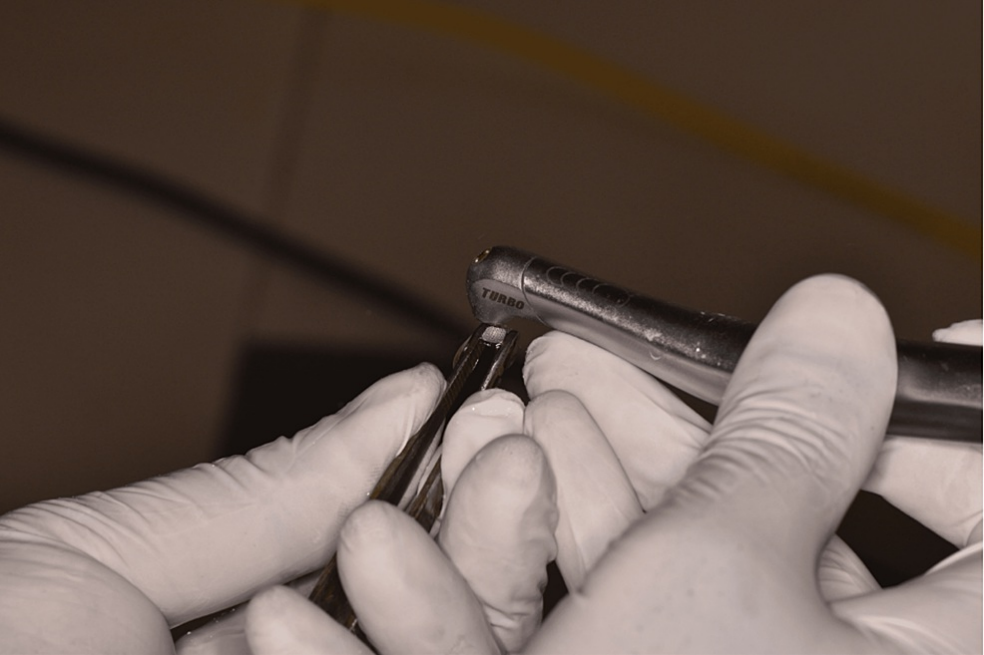
Laser treatment on bracket bases using Waterlase iPlus laser system

A Waterlase iPlus system was used for this study. This system is comprised of Er,Cr:YSGG lasers, which have been previously experimented with for various soft tissue and hard tissue applications. The study group brackets were subjected to laser irradiation by placing the brackets perpendicular to the laser direction, as depicted in Figure 1. The output of the laser was kept at a frequency of 50 Hz and power of 4.5 Watts in 'non-contact' mode with minimal water output at level 1 in order to prevent any major distortion of the bracket bases as given in Figure 1.

Surface analysis 

**Figure 2:**
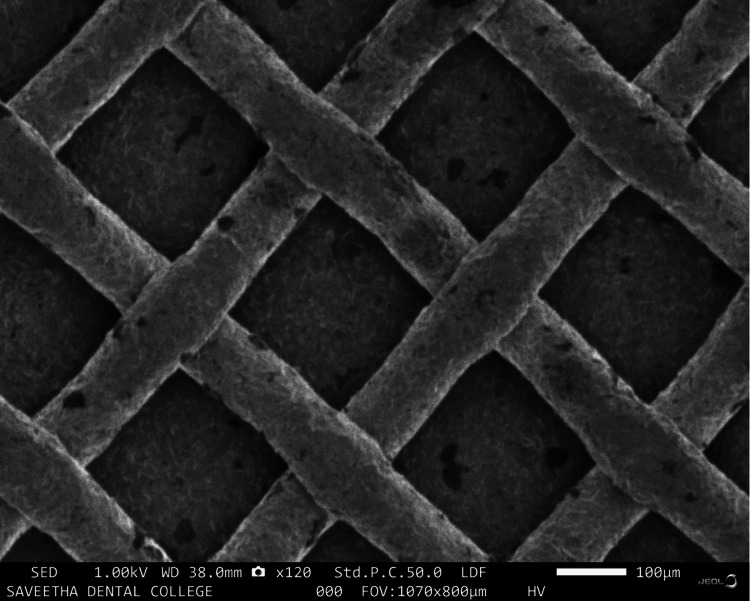
SEM analysis of laser treated bracket bases

**Figure 3:**
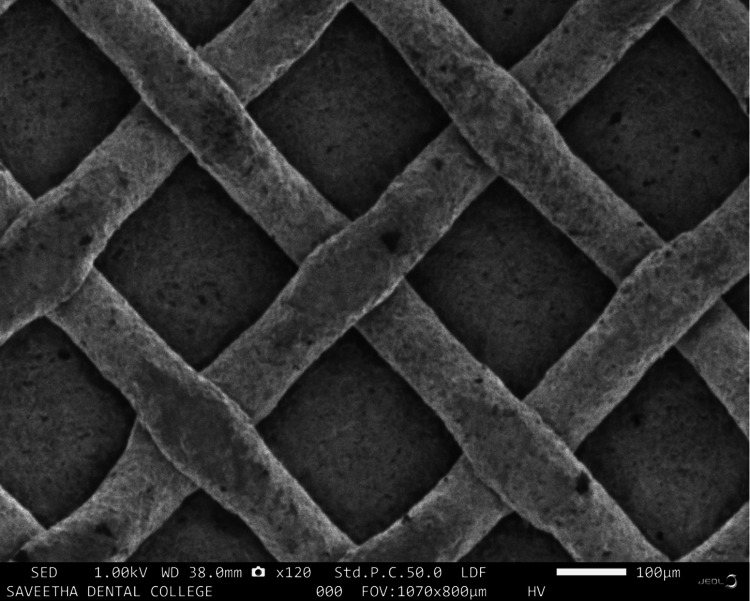
SEM analysis of untreated bracket bases

A comparison of surface alterations in the bracket bases after laser treatment was made by subjecting a single sample from each of the two groups to scanning electron microscope (SEM) analysis. The SEM images were as given in Figures 2-3. The laser-treated bracket bases had mild surface irregularities (Figure 2) when compared to the untreated bracket bases (Figure 3).

Teeth and bonding procedures

Twenty premolars were selected for the study, which were previously extracted for the purpose of orthodontic therapy. The teeth having intact buccal surfaces without any caries, fillings, or stains fulfilled the inclusion criteria and were selected for the study. The teeth were vertically embedded in self/cold cure acrylic resin until approximately 2 mm of the cemento-enamel junction was exposed. 37% orthophosphoric acid (prime etchant) was used to etch the enamel surface for 30 seconds, and the tooth surface was rinsed and dried. On the surface that was etched, a bonding agent (Transbond XT) was applied and was polymerised by LED Bluephase (Ormco Enlight composite) for a period of three seconds.

**Figure 4:**
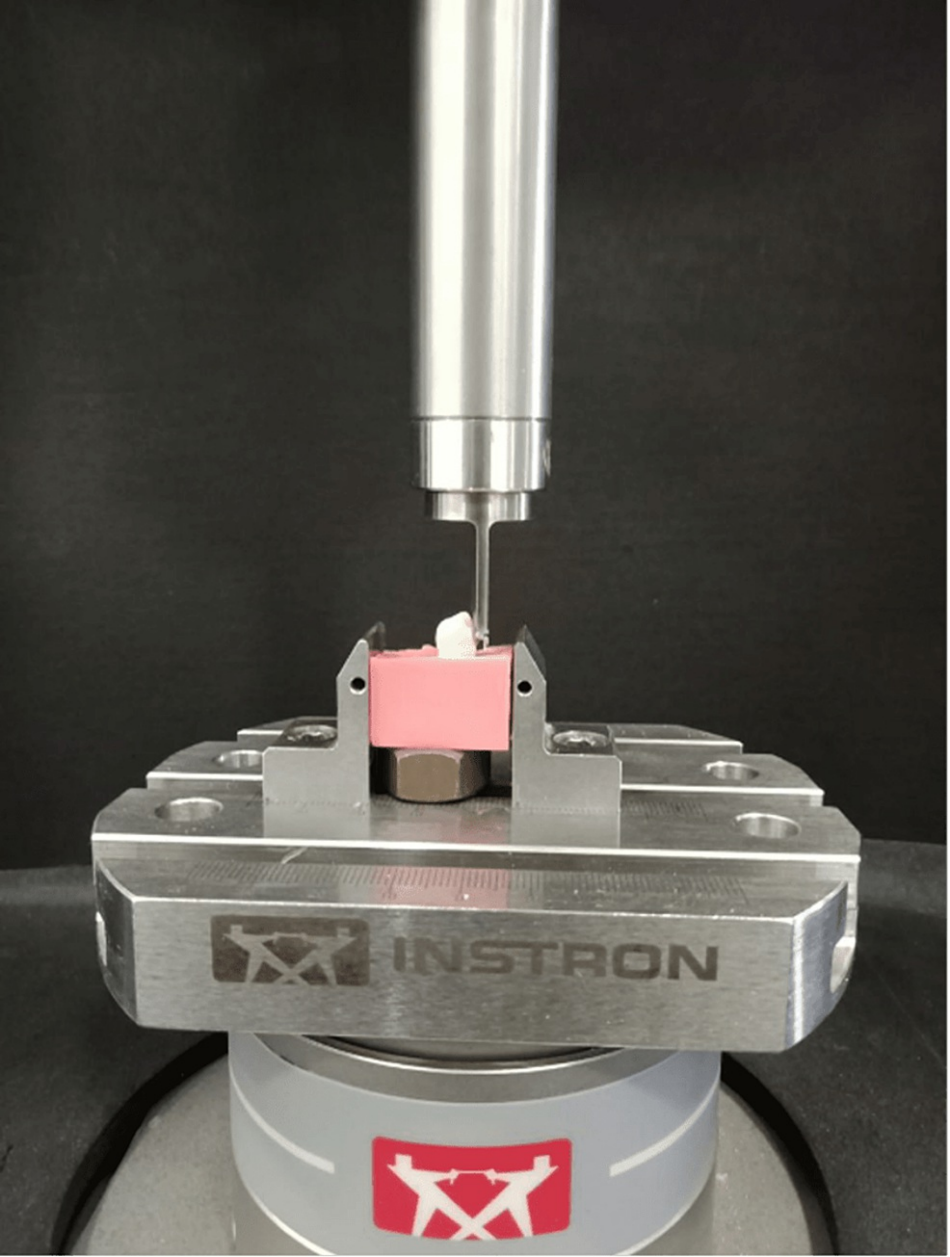
Shear bond strength of bonded brackets

Each of the brackets was physically placed on the labial surface of the premolars with the help of the composite. The extra resin was scraped off the sides, and the resin was then subjected to the LED Bluephase for polymerisation at a 300 mW/cm^2^ intensity and a distance of roughly 2 mm for five seconds on each side. These procedures were performed for all the brackets in both groups. A universal testing device (Instron E300 UTM) was used to measure the shear bond strength (SBS), as mentioned in Figure 4. During the SBS testing, each bonded tooth sample was placed in the loading apparatus with the labial surface placed parallel to the force. Each sample was subjected to a load that generated a shear bonding force at the enamel and bracket interface with the help of a shear blade with a dimension of 6.0 mm by 0.4 mm and a 100 kg load cell. The results were recorded in megapascals (MPa) on a computer that was connected to the universal testing device. The Adhesive Remnant Index (ARI index) was also measured after the debonding of the brackets based on the standard scoring criteria as mentioned in Table 1.

**Table 1:** Adhesive Remnant Index

Scores	Adhesive Remnant Index
0	Absence of adhesive on the enamel surface
1	<50% of adhesive remaining on the enamel surface
2	>50% adhesive remaining on the enamel surface
3	Entire adhesive remaining on the enamel surface

Statistical analysis

The IBM SPSS statistical software (version 23.1) was used to calculate the mean and standard deviation of the SBS of both groups. The data were normally distributed based on the results of the Shapiro-Wilk test, which was used to determine the data's normality. Hence, to determine the mean SBS for each group, an independent sample t-test was applied, and the results were obtained.

## Results

A total of 20 orthodontic brackets were evaluated for the present in vitro study, comprising 10 untreated brackets in the control group and 10 laser-etched brackets in the experimental group. The mean shear bond strength of control group brackets was found to be 8.63 ± 0.55 MPa. The mean shear bond strength of laser-etched brackets was 5 ± 0.40 MPa. The results stated that the control group brackets displayed stronger bonding efficiency than the laser-etched brackets (Table 2). But an independent sample t-test revealed that there was no significant difference between the two groups (p=0.23). The ARI scores for the control group brackets revealed that the majority of bond failures occurred at the interface between the enamel and the adhesive. The ARI scores of the laser-etched bracket group revealed that the bond failures occurred both at the interface between the enamel and adhesive and between the bracket and adhesive (Table 3).

**Table 2:** Descriptive analysis Level of significance (p<0.05)

Shear bond strength	Number of samples	Mean	Standard deviation	Significance (p-value)
Untreated bracket bases	10	8.63	0.55	0.23
Laser treated bracket bases	10	5	0.40	

**Table 3:** Adhesive Remnant Index scores (%)

Groups	0	1	2	3
Control (n=10)	0	7 (70)	3 (30)	0
Laser treated (n=10)	0	4 (40)	4 (40)	2 (20)

## Discussion

The SBS of orthodontic brackets is altered when bracket bases are exposed to laser radiation. When lasers were utilised in the surface modifications of the bracket base, some earlier tests claimed greater bond strength [7]. There have also been studies that have not shown any significant difference [8] or sometimes decreased SBS between laser-treated and untreated bracket bases [9,10]. According to Reimann et al., one of the key factors in the shear bond strength was the size and morphology of the bracket bases [8]. The curvature of the bracket base also needs to be effectively conformed to the tooth's surface. This criterion was better understood because of finite element analysis, which also showed that forces were distributed more uniformly across a tooth's surface the more the bracket base conformed to it. Shear bond strength can also vary depending on laser factors such as wavelength, frequency, power, and exposure of the bracket base surface. Another important factor is the application of a thin layer of resin between the enamel and base; otherwise, the interface may be weak due to the resin and its mechanical properties [11].

When laser-etched brackets were examined, Cozza et al. stated that they showed significantly lower bond strength values. Elsaka et al. stated that the manner of loading orthodontic brackets and the selection of orthodontic bracket materials could affect the bond strength of brackets [9,12]. According to Reynold et al., 6 to 8 MPa is the range for appropriate bond forces in orthodontics [13,14]. According to Kiryk et al., using the Erbium YAG laser in addition to traditional etching increases the bonding of various composites to tooth structures [15].

Er,Cr:YSGG lasers were mainly used for rebonding the debonded bracket bases and to etch the enamel surface. Abe et al. observed that the bond strength measured initially was substantially higher than the mean bond strength post-rebonding in their investigation [16]. The Er,Cr:YSGG laser demonstrated its potential to rebond ceramic brackets by successfully removing the remaining bonding material from the base of the brackets without interfering with its base surface [17]. According to Srivastava, laser etching was found to be more successful than acid etching, and both methods can be used to alter the bonding strength [18]. Ozer et al. stated that the tooth enamel was exposed to various irradiation powers using an Er,Cr:YSGG laser to prepare it for bonding and had discovered that irradiation with a 1.50-Watt laser caused enough etching for adequate bonding, but that 0.75-Watt laser irradiation had not yielded similar results [19]. Thus, the wattage of the laser irradiation also played a crucial role in altering the bond strength of the brackets. Bhagwan et al. discovered that laser irradiation at a power of 2 and 2.5 W for 10 seconds was more potent than acid etching and sufficient to etch enamel [20]. This result was in line with research by Usmez and Aykent [21] and Berk et al. [22], who compared the bonding efficiency using the Er,Cr:YSGG laser with a power of 1 Watt for a time period of 15 seconds and had discovered that it was substantially less than conventional acid etching [21,22]. In a study by Ahrari et al., the use of both sandblasting with aluminium oxide and the use of an Er,Cr:YSGG laser were effective in the study's conditions in removing the composite adhesive from bracket bases, and the bond strength of the rebounded brackets was dramatically reduced [23].

In recent years, with the emergence of hard tissue lasers available on the market that are relatively easy to source through a lab setup, the decision to individually laser treat bracket bases was initiated. This study was primarily undertaken to determine if this method of individualised laser treatment could increase the bond strength or have an inadvertent effect on the bracket base, thereby accidentally damaging the base of the orthodontic brackets and making the bond weaker than in the control group. The study's findings show that laser treatment of orthodontic bracket bases reduced the mean shear bond strength of the brackets. The study group brackets that had been laser-treated had surface irregularities, according to the SEM results. Though not statistically significant, the data of both groups revealed that the bond strength of untreated bracket bases was greater than that of laser-treated bracket bases when the bonded premolars were put through a shear bond strength test utilising the universal testing device. This could be due to the disruption of the mesh of the bracket bases due to the laser irradiation, which led to a decrease in SBS. As a result, all of the data from this in vitro study must only be viewed as assessments of the clinical outcomes and should be compared with those from other studies that followed similar standards.

Limitations

The limitations of the study included a decreased number of samples within each group. As this was an in vitro study, other causative factors and patient considerations could not be assessed, which could eventually lead to the bond failure of orthodontic brackets.

## Conclusions

The study's results indicated that laser therapy with Er,Cr:YSGG lasers applied to orthodontic bracket bases resulted in a reduction in bond strength. This effect could be attributed to the disturbance of the mesh structure within the bracket bases. The frequency, power, and exposure time of laser therapy are likely significant factors that influence the impact of lasers on orthodontic bracket bases. Therefore, further investigation is necessary to experiment with various frequencies and wattages to induce alterations in the bracket base, aiming to enhance the bond efficiency.
